# Cultivating Proactive Career Behavior: The Role of Career Adaptability and Job Embeddedness

**DOI:** 10.3389/fpsyg.2021.603890

**Published:** 2021-10-07

**Authors:** Peng Peng, Yu Song, Guangtao Yu

**Affiliations:** ^1^Business School, Central University of Finance and Economics, Beijing, China; ^2^School of Economics and Management, Southeast University, Nanjing, China

**Keywords:** career adaptability, career construction theory, regulatory focus, job embeddedness, proactive career behavior

## Abstract

Scholars have widely acknowledged that proactive career behavior is essential for individuals to proactively build their careers, as well as facilitate positive career outcomes. However, there are still many questions about how to activate proactive career behavior. In the current study, we consider whether, how and when regulatory focus of individuals would evoke their proactive career behavior. Based on career construction theory, we utilized the career adaptability framework to develop and test the mediating effect of individual regulatory focus on proactive career behavior through career adaptability. Moreover, we further proposed that job embeddedness plays a contingency role in moderating the extent to which regulatory focus contributes to proactive career behavior with the mediation of career adaptability differently and uniquely. Using a sample of 247 participants and collecting data in three waves, we found that the promotion focus of employees positively influences their proactive career behavior through the mediation of career adaptability. Furthermore, the indirect effect of promotion focus on proactive career behavior *via* career adaptability was moderated by the dichotomy of job embeddedness of individuals respectively and differently. Specifically, the positive relationship between promotion focus and proactive career behavior via the mediation of career adaptability was strengthened by the on-the-job embeddedness of employees, whereas the relationship was weakened by their off-the-job embeddedness. The overall findings broaden our understanding in terms of the underlying mechanism of proactive career behavior, suggesting that the promotion focus of individuals fosters proactive career behavior *via* career adaptability, and on-the-job and off-the-job embeddedness as contingency factors alter the effect of career adaptability.

## Introduction

In the volatile world of today, a boundaryless career, which is nonlinear and discontinuous, is vital to employee career management (Arthur and Rousseau, 1996; Wang and Wanberg, 2017; Guan et al., 2019), and scholars are increasingly paying more attention to how employees shape their careers individually and actively (Seibert et al., 2001; Strauss et al., 2012; Smale et al., 2019). Extant literature has shown that proactive career behavior, which refers to the concept that individuals will proactively explore options, set goals, build networks, and develop their skills and abilities to ensure the competitiveness of their career future (Claes and Ruiz-Quintanilla, 1998; Strauss et al., 2012), plays a key role in enabling individuals to proactively build their careers (Crant, 2000; Bindl and Parker, 2010; Parker et al., 2010), and contributes to positive individual career outcomes, such as career success (Eby et al., 2003; Forret and Dougherty, 2004; Zacher, 2014a), employment (Saks and Ashforth, 1997; Brown et al., 2006), and career satisfaction (Seibert et al., 1999, 2001).

However, considering the literature on proactive career behavior, we found that the antecedents and mechanisms of proactive career behavior of individuals require further examination. First, the antecedents and mechanisms of proactive career behavior require further study. The integrative mechanism of proactive career behavior has become an important topic of interest for understanding how and why individuals adopt proactive career behavior such as career planning, network building, or developing their skills (Strauss et al., 2012). However, prior research has been mainly focused on the dispositional antecedents of proactive career behavior such as personality (i.e., the Big Five traits and proactive personality) (Bateman and Crant, 1993; Hoyle and Sherrill, 2006; Teixeira et al., 2012; van Vianen et al., 2012), future work selves (Taber and Blankemeyer, 2015), self-efficacy (Hirschi et al., 2013), and hope and optimism (Luthans and Youssef, 2007). Considering that the consequences of proactive career behavior are often accompanied by risks (Ashford and Cummings, 1983; Ashford et al., 2003; Smale et al., 2019), prior research has unfortunately neglected contextual factors, which can also influence individual proactive career behavior significantly (Savickas, 2005, 2013; Ren and Chadee, 2017; Smale et al., 2019). Moreover, regarding the career adaptability conceptual model, scholars have highlighted the importance of studying career-adapting responses (e.g., proactive career behavior) from an interaction and contingency perspective (Rudolph et al., 2017). However, extant research has mainly focused on the dispositional antecedents of proactive career behavior, overlooking contextual factors that could be equally critical to determining adapting responses of individuals (Savickas, 2013; Rudolph et al., 2017).

Second, research on the relationship between career adaptability and related career behavior requires further exploration. Career adaptability is a core concept of the career construction theory (CCT), proposed by Savickas (2005, 2013), and has been tested worldwide, replacing the career maturity concept. Career adaptability refers to the psychosocial resources of individuals that enable them to conquer career challenges, transitions, or trauma (Savickas, 1997, 2005, 2013), and is vital for individuals to proceed with their adaptive career behaviors or outcomes. Empirical studies have focused on the desirable career-related outcomes of career adaptability, such as better performance, increased salary, and career satisfaction (Zacher, 2014b; Guan et al., 2015); however, they have overlooked the career behavior corresponding to career adaptability. Proactive career behavior is an important proactive behavior that is related to career adaptability and refers to actions undertaken by individuals to plan their careers, develop their skills, set goals, and accumulate experiences to ensure their future employability (Claes and Ruiz-Quintanilla, 1998; Strauss et al., 2012). Previous studies have explored the positive relationship between career adaptability and proactive career behavior (Strauss et al., 2012); however, since career adaptability is a core concept of the CCT (Savickas, 1997, 2005, 2013), studies that explore how the career construction conceptual model (Hirschi et al., 2015; Rudolph et al., 2017) enriches adaptability and adapting responses are scarce.

Lastly, while job embeddedness has been previously explored in literature, its critical effect on work-related behaviors has not been adequately studied. Job embeddedness refers to the “web of influences” that influence the intentions of employees to stay or leave their current employers (Mitchell et al., 2001; Felps et al., 2009). It can be categorized into on-the-job and off-the-job embeddedness. Prior research has shown how job embeddedness with organizations of individuals prevents them from leaving negative work situations (Allen et al., 2016), thereby alleviating the impact of job search on turnover (Swider et al., 2011). However, although prior research has laid a comprehensive foundation in examining the moderating effect of job embeddedness on employee work behaviors, on-the-job embeddedness and off-the-job embeddedness are theoretically distinct concepts (Porter et al., 2019) and, thus, have a different and respective influence on employee work behaviors. Unfortunately, limited studies have investigated the effects of this dichotomy of job embeddedness (Porter et al., 2019). Moreover, prior literature on job embeddedness has mainly focused on its beneficial aspects in terms of employee retention, neglecting its impact on proactive work behaviors. Empirical studies have shown that it is crucial to recognize the influence of job embeddedness in shaping individual withdrawal processes from organizations; however, it should be noted that it is much more important to stimulate proactive work behaviors of employees within an organization in this era of increased innovation and competition (Crant, 2000; Parker and Collins, 2010). Since job embeddedness and its effects on career behaviors of employees (i.e., job search, turnover) have been examined, we argued that it would be beneficial to examine the different and distinct influence of job embeddedness on the proactive career behavior of individuals.

**Figure 1:**
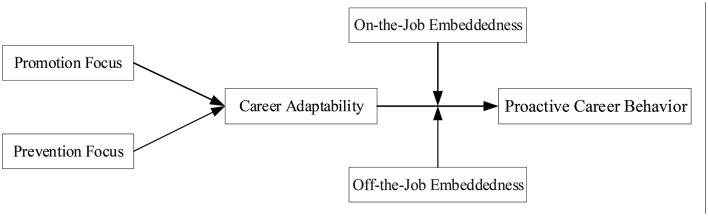
The hypothesized conceptual model.

We propose our study on extending the antecedents of proactive career behavior and enriching the understanding of its psychological mechanism based on the CCT (Savickas, 2002, 2005, 2013). We explored how and when the dichotomy of regulatory focus may lead to proactive career behavior. The CCT states that the career behaviors of individuals are constructed and shaped by their interactions with internal and external factors. An individual with a promotion focus is more concerned with accomplishments, hopes, and ambitions, whereas an individual with a prevention focus is more inclined toward safety, responsibilities, and obligations (Higgins, 1998, 2012). We argued that a promotion focus is positively related to proactive career behavior while a prevention focus is negatively related to proactive career behavior, moderated by career adaptability. Based on the CCT, we further proposed that the indirect effects of regulatory focus on proactive career behavior arising from career adaptability would be strengthened by on-the-job embeddedness and weakened by off-the-job embeddedness. In testing this proposition, we conducted a three-wave empirical study. Figure 1 depicts our theoretical framework.

This study contributes to the literature on proactive career behavior in two ways. First, we enriched the career construction model of adaptation by building linkages among adaptivity (i.e., regulatory focus), adaptability resources (career adaptability), contextual moderators (i.e., on-the-job and off-the-job embeddedness), and adapting responses (i.e., proactive career behavior). Our findings indicate that the regulatory focus of individuals plays an important role in triggering their beneficial career behavior. Second, we identify that on-the-job and off-the-job embeddedness, as core contingencies, influenced the proposed effects of regulatory focus through career adaptability differently and respectively.

## Theory and Hypothesis

### Career Construction Perspective on Proactive Career Behavior

The career construction theory (CCT) describes how individuals construct themselves, derive meaning from their careers, and adapt to their social environment with the goal of achieving a person-environment fit (Savickas, 2002, 2005, 2013). This theory adopts an integrative perspective wherein individuals must adapt to the expectations of their work and occupations (Strauss et al., 2012). Thus, the CCT provides a theoretical foundation for understanding why individuals gain new skills, develop their resources and abilities to influence their career outcomes, and guarantee future employment (Strauss et al., 2012, Savickas, 2013, Rudolph et al., 2017). Furthermore, this theory integrates vocational personality, career adaptability, and life themes to explain which, how, and why individuals are driven to enact vocational behaviors (Savickas, 2005). The theory states that career development is the product of individuals integrating their personal needs with psychosocial expectations to adapt to their environments. According to the guiding theoretical CCT framework, proposed by Rudolph et al. (2017), individuals differ in their willingness, which represents their adaptivity (adaptive readiness) in terms of their cognitive ability, big five traits, self-esteem, and adaptability resources, or their career adaptability in terms of their ability to develop beliefs and exhibit adapting responses (i.e., career planning, career exploration, and self-efficacy), thus leading to a positive fit, and integration with their work roles, which refer to adaptation results (i.e., job, career, school satisfaction, career identity, employability, and turnover intention) (Hirschi et al., 2015).

A regulatory focus refers to the orientation of an individual that guides his/her behavior; it comprises two kinds of focus: promotion focus and prevention focus (Higgins, 1997). Promotion focus is the orientation in respect of setting ideal goals, which are innovative and initiative (Johnson et al., 2017). Prevention focus is the orientation with regard to avoiding the negative outcomes, like risks, responsibility, and obligation, which aims to minimize loss and financial costs (Liang et al., 2012). From a CCT perspective, regulatory focus represents the stable propensity of individuals to undertake proactive career behavior. Regulatory focus refers to the willingness or beliefs, which individuals utilize to guide their behavior. A promotion focus drives individuals to focus on achieving positive career outcomes, whereas a prevention focus motivates individuals to pay attention to prevent the negative career outcomes from happening. From this perspective, regulatory focus can be viewed as adaptivity or adaptive readiness in the CCT theoretical framework.

Proactive career behavior refers to behaviors, such as career planning, building new networks, seeking advice, and acquiring skills, which individuals adopt to manage their careers (Strauss et al., 2012; Taber and Blankemeyer, 2015; Smale et al., 2019). According to Parker and Collins (2010), proactive career behavior is proactive “person-environment fit behavior,” which refers to the ability to enhance the fit of demand abilities and supplies values. Proactive career behaviors refer to behaviors wherein individuals take the initiative to build their careers rather than only respond to opportunities. They focus on long-term compatibility and harmony between the career expectations, orientations, and organizational requirements of individuals. From the perspective of the CCT theoretical framework, adapting responses entails adaptive behaviors and beliefs that individuals use to cope with career development tasks, as well as changing work and career conditions (Savickas and Porfeli, 2012; Hirschi and Valero, 2015; Hirschi et al., 2015; Rudolph et al., 2017). Proactive career behaviors involve individuals proactively dealing with their career development tasks through career planning, building new networks, seeking out advice, and acquiring skills. Thus, we assumed that proactive career behaviors serve as adaptive responses of individuals.

Different regulatory foci influence proactive career behaviors of individuals differently and separately. Employees with a promotion focus are more prone to display proactive career behaviors, whereas employees with a prevention focus are more vigilant of potential losses such that they are inclined to remain in their current states. According to the career construction model of adaptation, adaptivity or adaptive readiness influences adapting responses (Rudolph et al., 2017). Since regulatory foci are dichotomous and distinct, they (adaptivity or adaptive readiness) influence proactive career behaviors (adaptive responses) differently and individually. Specifically, employees with a promotion focus are more inclined to set ideal goals, be innovative, or take initiative such that, in terms of career management, they pay more attention to achieving positive career goals, thus enacting positive career behaviors that would benefit their careers (Strobel et al., 2017; Hulshof et al., 2020). Moreover, with regard to proactive work behavior, scholars have found that promotion focus has a positive effect on proactive strategic scanning (Strobel et al., 2017). In other words, since the consequences of proactive career behaviors are often accompanied by risk (Ashford and Cummings, 1983; Miller and Jablin, 1991; Ashford et al., 2003), individuals with a prevention focus, recognizing the risk of potential loss, are more vigilant to the negative consequences of proactive career behavior, to the point of being conservative or passive. Consequently, although they recognize the potential benefits, in the interest of averting potential losses or maintaining the status quo, individuals with a prevention focus avoid enacting proactive career behaviors and remain tacit. Promotion and prevention foci strengthen the relationship between psychological safety and using the voice of one differently and respectively (Song et al., 2020). Prior studies have shown that regulatory foci of individuals affect work-related outcomes such as task performance, organizational citizenship behavior (OCB), innovative performance, as well as work engagement (Liang et al., 2012). Thus, we proposed Hypotheses 1a and 1b as follows:

*Hypothesis 1a*: Promotion focus is positively related to proactive career behavior.
*Hypothesis 1b*: Prevention focus is negatively related to proactive career behavior.

### The Mediating Role of Career Adaptability

Career adaptability is a pivotal concept of the career construction theory (CCT) and refers to the capacities, competencies, and resources of individuals in terms of confronting the traumas, transitions, and vocational development problems faced in their careers (Savickas and Porfeli, 2012; van Vianen et al., 2012; Maggiori et al., 2017). Career adaptability is essential for individuals to develop and determine the optimal strategy to guide their adaptive behaviors, which refer to adaptive responses in the CCT theoretical framework (Rudolph et al., 2017; Johnston, 2018). Extant studies have established career adaptability as multidimensional and hierarchical, both theoretically and empirically (Savickas, 2005, 2013; Rudolph et al., 2017). Career adaptability comprises four dimensions that contribute to self-regulation strategies: concern, control, curiosity, and confidence (Savickas and Porfeli, 2012; van Vianen et al., 2012; Maggiori et al., 2017; Yu et al., 2019). *Concern* refers to the extent to which an individual pays attention to and prepares for the vocational future on his/her own. *Control* indicates the beliefs that drive the responsibility of individuals to prepare for their careers and take control of their vocational situations and future. *Curiosity* reflects a tendency as well as the ability of an individual to explore professional environments such as developing and enriching work and vocational opportunities. *Confidence* refers to the self-efficacy with which individuals solve challenges or problems and their capacities to overcome barriers or obstacles confronted in professional activities (Savickas, 2005; Savickas and Porfeli, 2012).

Regulatory foci refer to the approaches or strategies that drive the behaviors of individuals (Higgins, 1998, 2012). According to regulatory focus theory, regulatory focus involves dichotomy, which includes the promotion focus and prevention focus (Higgins, 1998, 2012), promotion focus regulates individuals to be more inclined to set goals with regard to aspirations and accomplishments, whereas prevention focus regulates individuals to be more inclined to set goals referring to responsibilities and safety. In contrast, individuals with a high-promotion focus would be more vigilant in terms of the presence or absence of positive future outcomes such as innovation or promotion, while individuals with a high-prevention focus would be more vigilant to the presence or absence of negative outcomes such as failure or risk. A promotion focus regulates the hopes and aspirations functioning like setting maximal goals; however, a prevention focus regulates the duties and obligations functioning like setting minimal goals (Brendl and Higgins, 1996; Higgins, 2012; Lin and Johnson, 2015). Career adaptability represents the resources or adaptabilities that individuals perceive they need to conquer future vocational barriers or obstacles; therefore, individuals with a high-promotion focus are more eager to set ideal and maximal goals due to the competitiveness for their future careers, i.e., to make the desired outcome happen (van Vianen et al., 2012; Direnzo et al., 2015; Li et al., 2015; Corr and Mutinelli, 2017; Guan et al., 2017). In contrast, individuals with a high-prevention focus are less likely to change; they are more inclined to set minimal goals to maintain the status quo to avoid undesired outcomes (Higgins, 2005; van Vianen et al., 2012; Li et al., 2015; Guan et al., 2017; Verbruggen and De Vos, 2020). Therefore, we expected that individuals with a high-promotion focus would be more concerned about their career development, more eager to control their careers, more curious about new career opportunities, and more confident about their ability to conquer future challenges. In contrast, individuals with a high-prevention focus are less likely to change, consider fewer alternatives (van Vianen et al., 2012), less concerned about future career development, believe they have less control over their careers, less curious about new career opportunities, and less confident about their ability to overcome future challenges.

According to the CCT, adapting to the environment of one with the goal of achieving person-environment integration is the driving force behind human development (Savickas, 2005). To adapt means to fit in, which entails a sequence that involves adaptive *readiness*, adaptability *resources*, adapting *responses*, and adaptation *results* (Savickas and Porfeli, 2012). Regulatory focus is the factor that regulates individuals to differ in their willingness to adapt; from this point of view, it is defined as adaptivity (adaptive readiness). Career adaptability refers to the general capacities, competencies, and resources that are essential for adapting to changing conditions (adaptability resources). The CCT theoretical framework states that adaptivity or adaptive readiness influences adaptability resources (career adaptability), aggregated along four dimensions (concern, control, curiosity, and confidence). Considering career adaptability is not only a perception of the current situation but also a reflection of the future (van Vianen et al., 2012), individuals with different propensities regarding the future such as promotion focus and prevention focus would be different in coping with tasks, transitions, and traumas they will encounter in the future of their careers. Therefore, from a future-oriented perspective, individuals with high-promotion focus are more eager to achieve future success, while individuals with high-prevention focus are more vigilant in preserving the status quo. Thus, we proposed Hypotheses 2a and 2b:

*Hypothesis 2a:* Promotion focus is positively related to career adaptability.
*Hypothesis 2b:* Prevention focus is negatively related to career adaptability.

According to the CCT, career adaptability (adaptability resources) helps individuals to establish the strategies that direct adaptive behaviors of individuals (adapting responses). Individuals with high-career adaptability, as indicated by the four dimensions, would be more willing and prepared to deal with transitions, traumas, and complex problems in their vocational future. Therefore, career adaptability helps individuals to shape their characteristic style of adaptive responses (Savickas and Porfeli, 2012). Moreover, career adaptability refers to the integration of person-environment fit resources. It shapes the self-extension of individuals into their social environment since career adaptability governs the function of behaviors, which include orientation, inquiry, establishment, management, and secession (Savickas and Porfeli, 2012). Thus, career adaptability comprises strategies and actual adaptive behaviors aimed at achieving adaptation goals.

Proactive career behaviors refer to behaviors that individuals undertake to enhance their person-environment fit. They focus on long-term compatibility between individual attributes and organizational requirements (Strauss et al., 2012). Proactive career behaviors help individuals to take the initiative to further develop their careers rather than merely reacting to opportunities, aiming to achieve a better person-organizational situation in a self-initiated way. From this perspective, career adaptability refers to the adaptability resources that an individual relies on to manage changes in his/her career, thus result in adapting behaviors that help to achieve their adaptation goals. According to the CCT, individuals with high-adaptability resources would be more prone to demonstrating their career behaviors (e.g., career planning, network building, skill development, and career consulting) proactively to achieve a better long-term person-environment fit. Prior research has shown the positive relationship between career adaptability and proactive career behavior (Taber and Blankemeyer, 2015). Thus, based on the above discussion, we proposed Hypothesis 3:

*Hypothesis 3*: Career adaptability is positively related to proactive career behavior.

Regulatory focus is a stable, trait-like psychological characteristic that enables individuals to focus on long-term goals, hopes, duties, and obligations (Liberman et al., 2007; Higgins, 2012). In other words, it refers to the orientation or disposition of an individual, which regulates his/her behavior (Higgins, 1997). Career adaptability is a reflection of both the present and the future (van Vianen et al., 2012) and differs for individuals with different propensities regarding the future such as promotion focus and prevention focus. A promotion focus refers to setting maximal goals for the future, while a prevention focus refers to setting minimal goals aimed at preserving the status quo (Brendl and Higgins, 1996; Koopmann et al., 2019). These different foci will have varying impacts on career adaptability, which represents the resources or readiness of individuals to cope with transitions, traumas, and ill-defined problems successfully throughout their careers (Savickas and Porfeli, 2012). In contrast, proactive career behavior often results in risky consequences (Ashford et al., 2003). Individuals with a promotion focus are more likely to recognize future beneficial outcomes and undertake innovation and exhibit initiative rather than those with a prevention focus, which aims at preserving current conditions and avoiding potential loss.

Proactive career behaviors involve behaviors that reflect the initiative of an individual in career planning, network building, career consulting, and acquiring relevant skills (Strauss et al., 2012; Taber and Blankemeyer, 2015). Proactive career behaviors, such as career planning, indicate adapting responses since individuals use such behaviors to cope with career development tasks, as same as changing work and career conditions (Hirschi et al., 2015).

Based on the career construction model of adaptation of Rudolph et al. (2017), we assumed that career adaptability mediates the relationship between regulatory focus and proactive career behavior. Specifically, career adaptability has a mediated effect on the positive relationship between promotion focus and positive career behavior, while the mediation effect of career adaptability occurs in the negative relationship between prevention focus and positive career behavior. The career construction model of adaptation proposes that career adaptability is positively related to adapting responses and mediates the relationship between adaptivity (adaptive readiness) and adapting responses (Savickas and Porfeli, 2012; Rudolph et al., 2017). Therefore, consistent with the career construction model of adaptation, chronic, dispositional adaptivity (adaptive readiness) affects adaptability resources or capabilities (career adaptability), thus promoting adapting responses (career behaviors and beliefs). Prior research has shown that career adaptability mediates the association between adaptivity, which is indicated by core self-evaluations, proactive personality, and adaptative responses (i.e., career planning, decision-making difficulties, exploration, and self-efficacy) (Hirschi et al., 2015). Thus, we propose Hypotheses 4a and 4b as follows:

*Hypothesis 4a:* Promotion focus will have a positive indirect effect on proactive career behavior *via* career adaptability.
*Hypothesis 4b:* Prevention focus will have a negative indirect effect on proactive career behavior *via* career adaptability.

### The Moderating Roles of On-the-Job and Off-the-Job Embeddedness

Job embeddedness refers to the concept of “web of influences,” which represents individuals who feel stuck in their organizations and communities (Mitchell et al., 2001; Porter et al., 2019). Over the years, job embeddedness has been a focal means to understand why people decide to stay or leave their jobs (Felps et al., 2009; Rubenstein et al., 2019). Job embeddedness can be described as associations of an individual with his/her organization and community, which can be categorized as on-the-job and off-the-job embeddedness. There are three main critical aspects of both on- and off-the-job embeddedness: (1) “Links” refers to the extent to which an individual has formal or informal associations with other people or activities on a job or community. The stronger the links, the more an individual is tied to an organization or community. (2) “Fit” refers to the extent to which a job or community is compatible or comfortable with the perception of an individual of a job, organization, or surrounding environment. The better the fit, the higher the possibility that an individual will feel professionally and personally tied to an organization or community. (3) “Sacrifice” refers to the ease of the links that an individual would have to give up if he/she leaves the organization. The higher the sacrifice, the more difficulty the individual would face in quitting an organization or community (Mitchell et al., 2001; Lee et al., 2004; Felps et al., 2009). Job embeddedness refers to the accumulated group of influences that are generally noneffective but get people stuck in the state quo (Mitchell et al., 2001); it is theorized to directly influence the individual retention within the organization and shape the withdrawal process (William Lee et al., 2014), the anti-withdrawal process (Sekiguchi et al., 2008). In terms of the interaction effects of job embeddedness, research has shown that job embeddedness mitigates the withdrawal process of “shocks” on turnover (Holtom and Inderrieden, 2006), a job search on turnover (Swider et al., 2011).

However, it is worth noting that some prior studies regarded job embeddedness as a singular construct, neglecting the different effects of on-the-job and off-the-job embeddedness (Feldman et al., 2012; Porter et al., 2019). Recognizing the differences in the effects of on-the-job and off-the-job embeddedness, it was necessary to investigate the contingency moderation effect of on-the-job and off-the-job embeddedness. Moreover, on-the-job and off-the-job embeddedness has been found to influence employee outcomes, such as turnover (Lee et al., 2004; William Lee et al., 2014), in- and extra-role performances (Lee et al., 2004), attitudes (William Lee et al., 2014), and OCBs (Sekiguchi et al., 2008; Burton et al., 2010), differently.

On-the-job and off-the-job embeddedness act as a moderator in the CCT adaptation process. According to the CCT theoretical framework, adapting to the environment with the aim of person-environment integration is the driving force contributing to development (Savickas, 2005); the sequence of adapting process ranges over adaptive *readiness*, adaptability *resources*, adapting *responses*, and adaptation *results*. Since the CCT asserted that the person-environment integration forms the core of human development, contextual and situational factors that contribute to the adapting sequence should be considered (Rudolph et al., 2017). As previously discussed, we suggested regulatory foci as adaptive *readiness*, career adaptability as adaptability *resources*, proactive career behavior as adapting *responses*, while career adaptability mediates the association between regulatory foci and proactive behavior. Since job embeddedness entails the web of influences, which are accumulated, generally nonaffective that stuck people in the status quo (Mitchell et al., 2001), it refers to the contextual and situational factors contributing to the person-environment integration. Moreover, job embeddedness can be divided into two main categories: on-the-job and off-the-job embeddedness (Lee et al., 2004). Thus, in line with the CCT, we assumed that on-the-job and off-the-job embeddedness represents the collection of influences that embed an individual with his/her organization or community, which acts as the focal contingency moderator in the adaptive sequence of an individual.

On-the-job embeddedness has a synergic impact on the proactive career behavior of employees, specifically contributing to the positive relationship between career adaptability and proactive career behavior. On-the-job embeddedness (links, fit, and sacrifice) refers to the collection of influential factors that encourage employees to remain within their jobs and their organizations. Employees with high on-the-job embeddedness are involved in and enmeshed with people and projects within their organizations (i.e., links); they feel that their jobs/organizations can utilize their professional and occupational skills well (i.e., a good fit), and perceive that they will be sacrificing things they value if they quit (i.e., sacrifice). Employees with high on-the-job embeddedness may get more task-relevant information from colleagues (Seibert et al., 2001) and develop better networks with colleagues, which contributes to creating a great support system (Kwantes et al., 2007; Ng and Feldman, 2012a). This better information and coworker support would enhance the work-related resources of an employee within an organization to help him/her better deal with challenging tasks, or job/organizational situations. Since career adaptability refers to the resources which individuals accumulate to cope with transitions, traumas, and complex problems in their careers, employees with high on-the-job embeddedness tend to boost their resources to better cope with present and future tasks, thus exhibiting proactive career behavior aimed at finding an optimal person-environment fit in the long term (Bindl and Parker, 2010). Prior study has shown that on-the-job embeddedness positively influences work outcomes of individuals (e.g., job motivation, networking behavior, and organizational identification) (Ng and Feldman, 2014). Thus, based on the above discussion, we proposed Hypothesis 5a:

*Hypothesis 5a:* On-the-job embeddedness moderates the relationship between career adaptability and proactive career behavior such that higher on-the-job embeddedness enhances the positive effect of career adaptability on proactive career behavior.

Off-the-job embeddedness can reduce the proactive career behavior of employees, specifically mitigating the positive relationship between career adaptability and proactive career behavior. Employees with high off-the-job embeddedness feel tied down and stuck with the people and the activities within the community they live in (i.e., links); they feel that they and their families fit the community well and are satisfied with the community (i.e., fit), and they recognize that they will sacrifice valued things of the community if they leave (i.e., sacrifice). Employees with high off-the-job embeddedness may develop an affiliation with the community in which they live with their families. Employees who are increasingly embedded in the community are prone to continue participating and investing in community activities (Ng and Feldman, 2012b; Verbruggen and van Emmerik, 2020), establishing and maintaining a rich network of community contacts (Singh et al., 2018; Porter et al., 2019). Such investments may distract the resources of an individual into his or her job and organization. For instance, employees who participate in regular community activities may face a dilemma in attending network-building activities with colleagues. The more an employee invests in community activities, the lesser the resources he/she can invest in a job and organization. As per the reviewed literature, career adaptability refers to adaptive resources that help employees address complex problems that may arise in their careers. Employees with high-community embeddedness would have to spend their resources on the community links and activities, which may reduce their ability to take advantage of the adaptive resources to enact proactive career behavior, which aims at the long-term compatibility between individual attributions and organization requirements (Wells et al., 2008; Ng and Feldman, 2012b). Prior studies have shown that off-the-job embeddedness strengthens the positive relationship between informal job search and turnover decisions (Porter et al., 2019). Thus, we proposed Hypothesis 5b:

*Hypothesis 5b*: Off-the-job embeddedness moderates the relationship between career adaptability and proactive career behavior such that higher off-the-job embeddedness will attenuate the positive effect of career adaptability on proactive career behavior.

## Methods

### Ethics Statement

This study was conducted in accordance with the recommendations of the ethical guidelines set out by the ethical review board of the Central University of Finance and Economics. The study protocol was approved by the ethical review board of the Central University of Finance and Economics. All the subjects gave their written informed consent in accordance with the Declaration of Helsinki.

### Sample and Procedure

We collected data from full-time employees working in a construction company in Northern China. Before the survey started, we had sent a study announcement to all 293 full-time employees *via* email, and the employees were assured that their identities would remain anonymous; they should honestly complete the questionnaire within 20 min, and they would receive 5 RMB per wave for their participation. To ensure data confidentiality, we used a code on the questionnaires to link the three waves of surveys, and the questionnaires were accessible only to the authors.

Survey data were collected at three points. At Time 1, we sent questionnaires to 293 participants and asked them to report their demographics and regulatory foci. Two weeks later, at Time 2, the 268 participants who had responded in phase one were asked to rate their career adaptability and job embeddedness. At time 3, another 2 weeks later, the 252 participants who responded in both of the first two rounds were asked to describe their proactive career behavior.

Subsequently, the final sample comprised 247 valid responses, with an overall response rate of 84.30%. Although few participants dropped out in the second or third wave for reasons such as fatigue or busy work, a good response rate was achieved because senior management strongly encouraged employees to participate in all three waves of the survey. Furthermore, we performed a dropout analysis to examine whether the nonrespondents were selective (Goodman and Blum, 1996; Lance et al., 2000). Results revealed no significant differences between respondents and nonrespondents with respect to gender, $\chi_{\left(1\right)}^{2}$ = 0.03, *p* = 0.86, age, *t* (266) = 0.72, *p* = 0.47, education, *t* (266) = −1.49, *p* = 0.14, job title, *t* (266) = −0.49, *p* = 0.63, organizational tenure, *t* (266) = 0.52, *p* = 0.60, and length of employment, *t* (266) = 1.26, *p* = 0.21. Thus, the dropout seems to be nonselective.

Among the final sample, the average age of the participants was 34.16 years (SD = 7.94), 40.08% were female, 90.28% had a college degree, average organizational tenure was 5.19 years (SD = 5.70), and average working years was 9.98 years (SD = 8.33). The participants consisted of 159 employees (64.37%), 52 supervisors (21.05%), 33 middle managers (13.36%), and 3 top managers (1.21%).

### Measures

All the items in the survey had response options ranging from 1, *strongly disagree*, to 5, *strongly agree*. We conducted a translation and back-translation procedure using the established cross-cultural translation approach (Brislin, 1986).

### Regulatory Focus

We measured regulatory focus at Time 1. We used the 6-item scale of Higgins et al. (2001) to measure promotion focus, including “Compared to most people, are you typically unable to get what you want out of life?,” “Do you often do well at different things that you try?,” and “I feel like I have made progress toward being successful in my life.” The Cronbach's alpha coefficient was.78. We adapted a four-item scale from that of Higgins et al. (2001) to measure prevention focus^1^, including “Growing up, would you ever “cross the line” by doing things that your parents would not tolerate?,” “How often did you obey rules and regulations that were established by your parents?,” and “Growing up, did you ever act in ways that your parents thought were objectionable?” The Cronbach's alpha coefficient was 0.84.

### Career Adaptability

We used the 24-item career adapt-abilities scale (CAAS) of Savickas and Porfeli (2012) to measure the four dimensions of career adaptability (concern, control, curiosity, and confidence) at Time 2. The sample items were: “Thinking about what my future will be like,” “Taking responsibility for my actions,” “Investigating options before making a choice,” and “Taking care to do things well.” The Cronbach's alpha coefficient was.87.

### Job Embeddedness

We measured job embeddedness at Time 2. We used a 9-item scale of Felps et al. (2009) to measure on-the-job embeddedness. Sample items were: “My job utilizes my skills and talents well,” “I have a lot of freedom on this job to pursue my goals,” and “I am a member of an effective work group.” The Cronbach's alpha coefficient was 0.77. We adapted 9-item from that of Felps et al. (2009) to measure job embeddedness^2^. Sample items were: “I really love the place where I live,” “If I were to leave the area where I live, I would miss my neighborhood,” and “I participate in cultural and recreational activities in my local area.” Cronbach's alpha coefficient was.83.

### Proactive Career Behavior

We used the 13-item scale of Strauss et al. (2012) to measure the four dimensions of proactive career behavior (career planning, proactive skill development, career consultation, and network building) at Time 3. Sample items were: “I am planning what I want to do in the next few years of my career,” “I develop skills which may not be needed so much now, but in future positions,” “I seek advice from my supervisor(s) or colleagues about additional training or experience I need in order to improve my future work prospects,” and “I am building a network of contacts or friendships with colleagues to obtain information about how to do my work or to determine what is expected of me.” The Cronbach's alpha coefficient was 0.85.

### Control Variables

Following prior literature that emphasized the impact of tenure and work experience on proactive career behavior (Strauss et al., 2012; Smale et al., 2019), we controlled for organizational tenure, job title, and length of employment. We also controlled for the demographic factors of gender, age, and education level (Claes and Ruiz-Quintanilla, 1998; Hirschi and Freund, 2014).

## Results

### Descriptive Statistics

The descriptive statistics and zero-order correlations for our variables are shown in Table 1. The coefficient alphas are provided in parentheses on the diagonal.

**Table 1:** Means, SD, and correlations among the study variables.

**Variables**	**M**	**SD**	**1**	**2**	**3**	**4**	**5**	**6**	**7**	**8**	**9**	**10**	**11**	**12**
1. T1 GENDER	0.40	0.49	–											
2. T1 AGE	34.16	7.94	−0.02	–										
3. T1 EDU	3.33	0.69	−0.07	−0.18^**^	–									
4. T1 TITLE	1.51	0.77	−0.19^**^	0.45^***^	−0.12	–								
5. T1 TENURE	5.19	5.70	0.06	0.63^***^	−0.20^**^	0.1	–							
6. T1 WORK YEARS	9.98	8.33	−0.04	0.88^***^	−0.31^***^	0.42^***^	0.63^***^	–						
7. T1 PMF	3.77	0.56	−0.08	0.16^*^	−0.01	0.20^**^	0.19^**^	0.19^**^	(0.78)					
8. T1 PVF	3.85	0.80	0.06	0.05	0.08	−0.08	0.12	0.01	0.13^*^	(0.84)				
9. T2 CAA	3.90	0.51	−0.10	0.10	0.04	0.14^*^	0.13^*^	0.12	0.43^***^	0.06	(0.87)			
10. T2 NJE	3.76	0.58	−0.10	0.10	0.02	−0.01	0.14^*^	0.11	0.43^***^	0.16^*^	0.67^***^	(0.77)		
11. T2 FJE	3.41	0.77	−0.03	0.22^***^	−0.09	0.05	0.27^***^	0.21^**^	0.37^***^	0.25^***^	0.43^***^	0.54^***^	(0.83)	
12. T3 PCB	3.90	0.53	−0.01	0.07	−0.01	0.08	0.13^*^	0.10	0.46^***^	0.10	0.49^***^	0.44^***^	0.35^***^	(0.85)

*N = 247. Gender was coded as 0 = male, 1 = female. Education was coded as 1 = high school or below, 2 = junior college, 3 = undergraduate, 4 = graduate or above. Title was coded as 1 = employee, 2 = supervisor, 3 = middle manager, 4 = top manager. PMF, promotion focus; PVF, prevention focus; CAA, career adaptability; NJE, on-the-job embeddedness; FJE, off-the-job embeddedness; PCB, proactive career behavior*.

*T1, T2, and T3 refer to the time wave variables collected at phases 1, 2, and 3, respectively*.

*Cronbach α in parentheses*.

* *p < 0.05*,

** *p < 0.01*,

*** *p < 0.001*.

### Confirmatory Factor Analyses

We conducted confirmatory factor analyses (CFAs) using Mplus 7.4, considering the clustered nature of our sample and using robust maximum likelihood estimation. Our initial measurement model included six factors (i.e., promotion focus, prevention focus, career adaptability, on-the-job embeddedness, off-the-job embeddedness, and proactive career behavior). As Table 2 shows, this model was a good fit for the data: $\chi_{\left(476\right)}^{2}$ = 877.69, *p* < 0.001, comparative fit index (CFI) = 0.92, Tucker-Lewis index (TLI) = 0.91, root mean square error of approximation (RMSEA) = 0.06, standardized root mean square residual (SRMR) = 0.06. Next, we tested an alternative five-factor model. The only difference between this model and our first model was that, in this alternative model, we incorporated promotion focus and prevention focus as one factor. The fit of this model was significantly inferior to that of our proposed model, χ^2^_(481)_ = 1,063.11, *p* < 0.001, CFI = 0.88, TLI = 0.87, RMSEA = 0.07, SRMR = 0.06. Subsequently, we tested a four-factor model in which on-the-job and off-the-job embeddedness was considered as one factor. The fit of this model was also significantly inferior to that of our proposed model, $\chi_{\left(485\right)}^{2}$ = 1,146.98, *p* < 0.001, CFI = 0.80, TLI = 0.78, RMSEA = 0.09, SRMR = 0.08. Subsequently, we tested a three-factor model in which career adaptability and proactive career behavior were considered as one factor. The fit of this model was also significantly inferior to that of our proposed model, $\chi_{\left(489\right)}^{2}$ = 1,254.19, *p* < 0.001, CFI = 0.67, TLI = 0.64, RMSEA = 0.13, SRMR = 0.11. A final model in which all measures were loaded onto one factor also had a significantly inferior fit than our proposed model, $\chi_{\left(492\right)}^{2}$ = 1,642.19, *p* < 0.001, CFI = 0.55, TLI = 0.50, RMSEA = 0.15, SRMR = 0.12. Therefore, the CFAs indicated that our proposed six-factor model was a good fit for the data, and this fit was superior to that of simpler models. This result supported the validity of our specified measurement model.

**Table 2:** Results of confirmatory factor analysis of study variables.

**Model**	***χ*** **^2^**	***df***	***Δχ*** **^2^**	***Δdf***	**CFI**	**TLI**	**RMSEA**	**SRMR**
1.6-factor	877.69	476	–	–	0.92	0.91	0.06	0.06
2.5-factor	1,063.11	481	185.42	5	0.88	0.87	0.07	0.06
3.4-factor	1,146.98	485	83.87	4	0.80	0.78	0.09	0.08
4.3-factor	1,254.19	489	107.21	4	0.67	0.64	0.13	0.11
5.1-factor	1,642.19	492	388.00	3	0.55	0.50	0.15	0.12

*df, degrees of freedom; Δχ^2^, chi-square differences; Δdf, degrees of freedom differences; CFI, comparative fit index; TLI, Tucker-Lewis index; RMSEA, root mean square error of approximation; SRMR, standardized root mean square residual*.

*N = 247*.

*Model 1 (six-factor model) includes all study variables. Model 2 (five-factor model) combines on-the-job embeddedness and off-the-job embeddedness as one factor and considers the other four variables as four independent factors. Model 3 (four-factor model) combines on-the-job embeddedness and off-the-job embeddedness as one factor, promotion focus and prevention focus as one factor, and considers the other two variables as two independent factors. Model 4 (three-factor model) combines on-the-job embeddedness and off-the-job embeddedness as one factor, promotion focus and prevention focus as one factor, and career adaptability and proactive career behavior as one factor. Model 5 (one-factor model) combines all six variables as one factor*.

*Δχ^2^ and Δdf reflect differences of χ^2^ and df between the corresponding model and Model 1. All χ^2^ and Δχ^2^ are significant at the p < 0.001 level*.

### Test of Hypotheses

We tested all the hypotheses using structural equation modeling in Mplus 7.4. As summarized in Table 3, after including the controls, the promotion focus of employees was found to be positively related to career adaptability (*b* = 0.34, *p* < 0.001), thereby supporting Hypothesis 2a. Additionally, the relationship between promotion focus and proactive career behavior was significant (*b* = 0.26, *p* < 0.001), the relationship between career adaptability and proactive career behavior was significant (*b* = 0.40, *p* < 0.01), and the indirect effect of promotion focus on proactive career behavior mediated by career adaptability was significant (indirect effect = 0.13, *p* < 0.01); thus, career adaptability partially mediated the relationship between promotion focus and proactive career behavior, thereby supporting Hypotheses 1a, 3, and 4a. However, prevention focus was not significantly related to career adaptability (*b* = 0.00, *n.s*.) or proactive career behavior (*b* = 0.02, *n.s*.), and the indirect effect of prevention focus on proactive career behavior mediated by career adaptability was not significant (indirect effect = 0.00, *n.s*.). Therefore, Hypotheses 1b, 2b, and 4b were not supported.

**Table 3:** Summary of Hypotheses 1–4 results.

**Variables**	**Career adaptability**			**Proactive career behavior**		
	***b***	***SE b***	**β**	***b***	***SE b***	**β**
**Controls**						
Gender	−0.06	0.06	−0.06	0.04	0.06	0.05
Age	−0.01	0.01	−0.11	−0.00	0.01	−0.05
Education	0.04	0.05	−0.06	−0.02	0.04	−0.03
Title	0.05	0.04	0.08	−0.02	0.04	−0.03
Tenure	0.01	0.01	0.09	0.00	0.01	0.03
Working years	0.00	0.01	0.07	0.00	0.01	0.03
**Independent variable**						
Promotion focus	0.34	0.06	0.40^***^	0.26	0.08	0.23^***^
Prevention focus	0.00	0.04	0.01	0.02	0.04	0.03
**Mediator**						
Career adaptability				0.40	0.14	0.43^**^
**Indirect effect**						
Promotion focus → career adaptability → proactive career behavior				0.13^**^	0.06	
Prevention focus → career adaptability → proactive career behavior				0.00	0.02	
*R^2^*	0.20^**^	0.07		0.41^***^	0.09	

*N = 247*.

*Model fit statistics: χ^2^_(67)_ = 125.52, CFI = 0.95, TLI = 0.94, RMSEA = 0.06, SRMR = 0.04. b = unstandardized coefficient; SE, standard error; β, standardized coefficient*.

*Bootstrap = 5,000*.

** *p < 0.01,*

*** *p < 0.001*.

**Figure 2:**
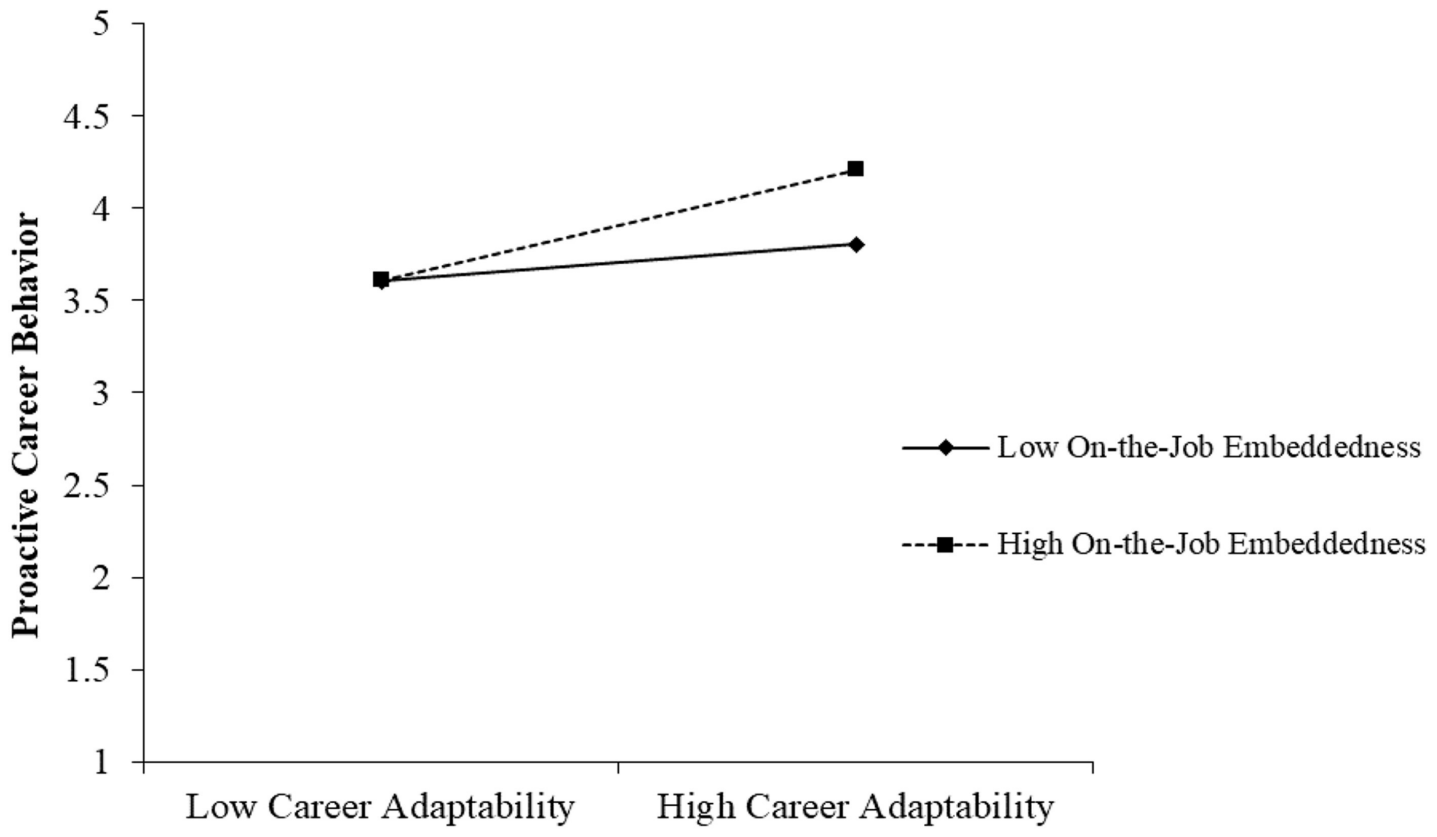
The effect of career adaptability on proactive career behavior at high and low levels of on-the-job embeddedness. The simple slope tests showed that the relationship between career adaptability and proactive career behavior was more positive for individuals with high on-the-job embeddedness (*b* = 0.59, *p* < 0.001) than for individuals with low on-the-job embeddedness (*b* = 0.20, *n.s*.).

Hypotheses 5a and 5b predicted that on-the-job and off-the-job embeddedness, respectively, of employees, would moderate the effect of career adaptability on proactive career behavior. Table 4 shows the results of these moderation effects. We found a positive interaction between career adaptability and on-the-job embeddedness regarding proactive career behavior (*b* = 0.40, *p* < 0.01). We plotted the relationships between career adaptability and proactive career behavior during high and low levels of on-the-job embeddedness (1 SD above and below the mean). As Figure 2 shows, the simple slope tests indicated that the relationship between career adaptability and proactive career behavior was more positive for individuals with high on-the-job embeddedness (*b* = 0.59, *p* < 0.001) than with low on-the-job embeddedness (*b* = 0.20, *n.s*.), meaning the relationship between career adaptability and proactive career behavior is stronger in the condition of high on-the-job embeddedness, thus supporting Hypothesis 5a.

**Table 4:** Summary of Hypotheses 5 results.

**Variables**	**Career adaptability**			**Proactive career behavior**		
	***b***	***SE b***	**β**	***b***	***SE b***	**β**
**Controls**						
Gender	−0.06	0.06	−0.06	0.06	0.06	0.07
Age	−0.00	0.01	−0.07	−0.00	0.01	−0.07
Education	0.04	0.05	0.06	0.03	0.04	0.04
Title	0.05	0.04	0.07	0.02	0.04	0.04
Tenure	0.01	0.01	0.09	0.00	0.01	0.00
Working years	0.00	0.01	0.03	0.00	0.01	0.05
**Independent variable**						
Promotion focus	0.36	0.06	0.40^***^	0.14	0.06	0.17^**^
Prevention focus	0.00	0.04	0.00	0.00	0.03	0.00
**Mediator**						
Career adaptability				0.33	0.08	0.38^***^
**Moderator**						
On-the-job embeddedness				0.08	0.06	0.11
Off-the-job embeddedness				0.07	0.04	0.13^†^
**Interaction term**						
Career adaptability × On-the-job embeddedness				0.40	0.09	0.55^**^
Career adaptability × Off-the-job embeddedness				−0.15	0.07	−0.21^*^
*R^2^*	0.20^**^	0.06		0.53^***^	0.07	

*N = 247*.

*The moderation and moderated mediation hypotheses were tested simultaneously. All continuous predictors were mean-centered (Edwards and Lambert, 2007)*.

*Model fit statistics: χ^2^_(127)_ = 236.84, CFI = 0.93, TLI = 0.91, RMSEA = 0.06, SRMR = 0.04*.

*b, unstandardized coefficient; SE, standard error; β, standardized coefficient*.

*Bootstrap = 5,000*.

† *p < 0.10*,

* *p < 0.05*,

** *p < 0.01*,

*** *p < 0.001*.

**Figure 3:**
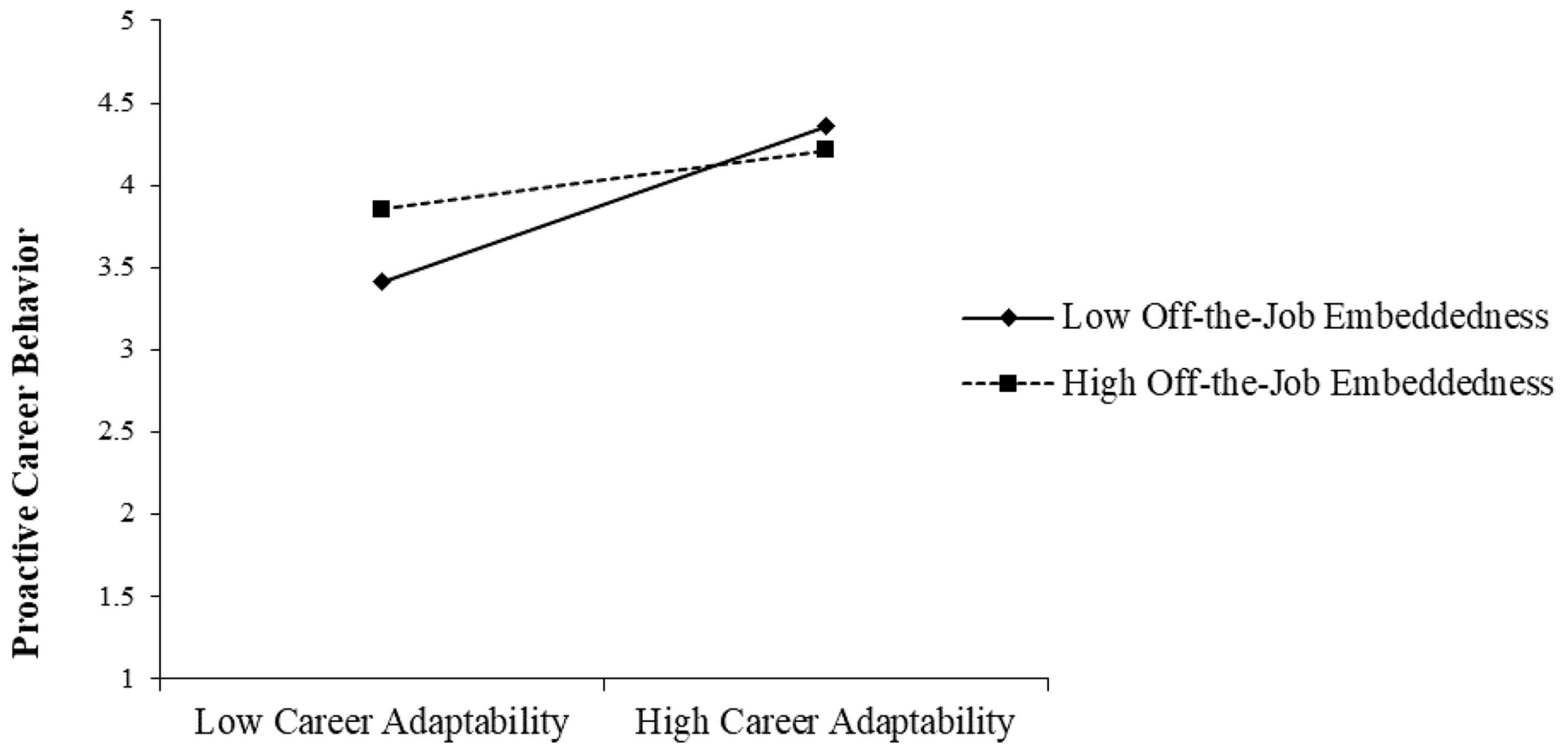
The effect of career adaptability on proactive career behavior at high and low levels of off-the-job embeddedness. The simple slope tests showed that the relationship between psychological safety and prohibitive voice behavior was more positive for individuals with low off-the-job embeddedness (*b* = 0.44, *p* < 0.001) than for individuals with high off-the-job embeddedness (*b* = 0.22, *n.s*.).

Additionally, the impact of the interaction between career adaptability and off-the-job embeddedness on proactive career behavior was significantly negative (*b* = −0.15, *p* < 0.05). We plotted the relationship between career adaptability and proactive career behavior at both high and low levels of off-the-job embeddedness (1 SD above and below the mean). As Figure 3 shows, the simple slope tests indicate that the relationship between career adaptability and proactive career behavior was less positive with high off-the-job embeddedness (*b* = 0.22, *n.s*.) than with low off-the-job embeddedness (*b* = 0.44, *p*< 0.001), meaning the relationship between career adaptability and proactive career behavior was weaker in the high off-the-job embeddedness situation, thus supporting Hypothesis 5b.

## Discussion

### Theoretical Implications

This study makes three contributions to the field of career management. First, our research provides new insights into why and how employees adopt proactive career behavior. It elaborates the impact of regulatory focus on proactive career behavior as well as offers a career adaptability mechanism to understand its influence. Particularly, we elaborated the CCT (Savickas, 2013; Rudolph et al., 2017) by building the linkages among adaptivity (i.e., regulatory focus), adaptability resources (i.e., career adaptability), contextual moderators (i.e., on-the-job embeddedness, off-the-job embeddedness), and adapting responses (i.e., proactive career behavior). The findings of this study are in line with the study which asserts that future work self-influences the proactive career behavior with the mediation of career adaptability dimensions (Taber and Blankemeyer, 2015), as well as the prior study pointing that regulatory focus of individuals would influence the proactive work behavior (Parker et al., 2010; Strobel et al., 2017). Researchers of proactive career behavior have long assumed that proactive career behavior of employees is derived from psychological traits of individuals, such as proactive personality, and possible self (Claes and Ruiz-Quintanilla, 1998; Strauss et al., 2012; Hirschi and Freund, 2014). However, prior studies have paid much attention to the disposition antecedents of proactive career behaviors, neglecting the integrated and mechanical system beneath proactive career behavior. By demonstrating that the promotion focus of employees facilitates proactive career behavior through career adaptability, this effect is moderated by on-the-job and off-the-job embeddedness, respectively. Our work explored how career adaptability is activated by regulatory focus, with the contingency effect of on-the-job and off-the-job embeddedness to systematically foster proactive career behavior; our results contribute to extend our current understanding of the underlying mechanisms of proactive career behavior and broaden the new vision to acknowledge the mechanism in predicting proactive career behavior with interaction and contextual way. Future research is called for to fully understand and enrich the antecedents and contextual factors contributing to proactive career behavior by adopting the conceptual framework of career construction theory.

Second, our study contributes to fully understand the relationship between career adaptability and proactive career behavior. Extant research has been focused on desired career-related consequences of career adaptability (Zacher, 2014b; Guan et al., 2015), yet overlooking the career behavior arising from career adaptability. The findings of our study are in line with the literature (Strauss et al., 2012), as well as clarifying the relationship between career adaptability and positive career behaviors by adopting the framework of CCT (Savickas, 2005, 2013; Rudolph et al., 2017). Our study further enriches the career adaptability and adapting responses; future research should pay more attention to the positive career behaviors related to career adaptability in the career management field. For example, job search success is critical for an individual to achieve long-term career success (DiPrete and Eirich, 2006; Guan et al., 2013). Career adaptability enriches individuals to possess adequate psychological and social resources to cope with career traumas and transitions to attain their career development; thus, career adaptability would facilitate job search success.

Third, one of the most significant contributions of this study is that we identified the role of on-the-job and off-the-job embeddedness as key contextual contingencies in cultivating proactive career behavior. Interestingly, we found that the ways in which career adaptability relates to proactive career behavior differ depending on the job embeddedness of the individuals. Specifically, for individuals with high on-the-job embeddedness, the effect of career adaptability on proactive career behavior was significantly strengthened. In contrast, for individuals with high off-the-job embeddedness, the effect of career adaptability on proactive career behavior was significantly reduced. This knowledge contributes to our understanding of how to intensify the activating effect of career adaptability on proactive career behavior. In prior pieces of literature, although (Strauss et al., 2012) examined the positive implications of cognitive representations of individuals (i.e., the interaction effect of future work self-salience and elaboration) for proactive career behavior, they did not explore how contextual factors affect its activating effect. To extend this line of research, we not only examined the mediating role of career adaptability in the relationship between regulatory focus and proactive career behavior but also demonstrated that on-the-job embeddedness of individuals strengthens the positive effect of career adaptability on proactive career behavior, while off-the-job embeddedness reduces the positive effect of career adaptability on proactive career behavior. This knowledge helped us to understand how web sticking of individuals (i.e., job embeddedness) affects the positive influence of career adaptability on proactive career behavior. In addition, our findings extend and enrich the contingency contextual factors in terms of job embeddedness, which systematically generate proactive career behavior. Prior studies mainly focused on the beneficial aspects of job embeddedness for organizations to keep employees stable in their positions (Mitchell et al., 2001; Felps et al., 2009); few studies have explored the effects with regard to two dichotomies of job embeddedness (Porter et al., 2019). Moreover, our findings are in line with the prior study that job embeddedness would influence proactive work behaviors of individuals such as networking (Ng and Feldman, 2014). According to the conservation of resources (COR) theory, prior studies have proposed that individuals are motivated to accumulate resources to lessen their potential losses in the future, despite the fact that their resources are sufficient at the present time (Hobfoll, 1989; De Cuyper et al., 2012); individuals who are more embedded within an organization and community would be more inclined to acquire resources in the work domain and, thus, exhibit proactive work behaviors (Ng and Feldman, 2014). In the CCT framework, our findings not only supported the previous notion that job embeddedness influences the proactive work behaviors of the individuals but also with a contextual view to shed light on a new direction to identify the relationship between job embeddedness and proactive work behaviors. Our study has initiated the discussion about exploring the respective contingency effects of on-the-job and off-the-job embeddedness on proactive career behavior, thus with study to illustrate the significant interaction in predicting proactive career behavior conceptually and empirically. In line with the career construction theory, the contextual factors, such as perceived organizational support (Abou Hashish, 2015), ethical work climate (Abou Hashish, 2015), job support (Lecca et al., 2020), excessive workload (Kowalczuk et al., 2020), and workplace incivility (Cash et al., 2019), would regulate the relationship between career adaptability and proactive career behavior, yet few studies have examined the influences of such contextual factors in facilitating or withdrawing the process. Herein, we call for more pieces of research aiming to understand the boundary conditions of career adaptability predicting proactive career behavior.

### Practical Implications

Since proactive career behavior is beneficial for individuals to initiatively build their careers (Crant, 2000; Bindl and Parker, 2010; Parker et al., 2010), as well as leading to career success (Eby et al., 2003; Forret and Dougherty, 2004; Zacher, 2014a), acknowledging the antecedents and mechanism in cultivating proactive career behavior has clear significance in the practical field. First, our findings have important implications for managers who seek to foster career adaptability and proactive career behavior of employees. Our study shows that a promotion focus effectively promotes career adaptability of employees and, thus, proactive career behavior. This finding can serve as a suggestion for organizations that managers should be aware of employees who possess a promotion focus. Compared with a prevention focus, the promotion focus is mainly concerned with the accomplishments, hopes, and ambitions that regulate the presence and the absence of positive outcomes and facilitate prosperity (Higgins, 1998). This indicates that individuals with a promotion focus can enhance their career adaptability, leading them to undertake proactive career behaviors to prepare for the future. In the modern era featuring boundaryless careers (Wang and Wanberg, 2017; Guan et al., 2019), it is more and more important for individuals to proactively build their careers and, thus, achieve their career success and satisfaction. Our findings suggest that practitioners should pay much attention to foster employees with the promotion focus to energize the organizational human resources effectiveness. Moreover, since career adaptability enables individuals to cope with traumas and transitions of their careers (Savickas, 2013), employees with promotion focus would be more prone to accept challenges and difficulties arranged by an organization, such as being an expatriate to work overseas, exploring and developing emerging markets.

Second, organizations must also be aware of the impact of the job embeddedness of employees. Our findings regarding the moderating roles of two dichotomies refer to job embeddedness of employees, that is, the effect of career adaptability on proactive career behavior is amplified by on-the-job embeddedness of individuals, mitigated by off-the-job embeddedness. Thus, it seems relevant for HR managers and leaders to pay attention to the underlying reasons for on-the-job and off-the-job embeddedness, and how to convert off-the-job embeddedness into on-the-job embeddedness, such as by jointly participating in corporate social responsibility projects with communities to improve the relationship between employees and organizations. In addition, practitioners should be well acknowledged with the beneficial aspects of on-the-job embeddedness in shaping the effective career path for employees to achieve career success. By increasing the on-the-job embeddedness of an employee, with the ways of facilitating work group building, enhancing the fit with work and organization, carrying out long-term employee incentive projects, such as stock ownership, internal saving plans, housing assistance schemes, and retention bonuses, proactive career behavior of employees would be facilitated and thus reinforce the organizational career management effectiveness.

### Limitations

This study has several limitations. First, although our CCT-based study elucidates how regulatory focus (i.e., adaptivity) affects proactive career behavior (i.e., adapting responses) *via* the mediating processes of career adaptability (i.e., adaptability resources) and the moderating process of job embeddedness, we recognize that distal consequences (i.e., adaptation results) were not included in our research model. It should be noted that the aim of career adaptation is to align the personal needs of individuals with environmental demands and opportunities. Adaptation results in terms of the goodness of fit between a worker and his/her environment, as well as other indicators, such as satisfaction, development, and work success (Savickas and Porfeli, 2012; Savickas, 2013; Rudolph et al., 2017), are beneficial to be examined in the CCT-based study. We realized that one of our limitations is that we did not examine the CCT framework comprehensively, neglecting the adaptation results of the regulatory focus.

Second, it is important to acknowledge that our research sample comprised only one Chinese company, which leads to some limitations of this study. On one hand, Chinese people are directed by the Confucian value of harmony, which means that Chinese employees are more prone to express their hopes rather than their worries (Huang et al., 2005; Guan et al., 2018). On the other hand, even though the construction industry is one of the most popular industries in China, the scope of this study is limited by one Chinese constructive company. Thus, another limitation of this study is that we did not examine our hypotheses in a multi-culture and multi-industry way.

Third, we collected our longitudinal data by three waves; however, it is worth noting that our participants rated the scales by self-report. Thus, by recognizing the way self-report still needs improvements in multisource data collection, our findings with regard to the mechanism of regulatory focus contributing to proactive career behavior have their limitations; future research should apply multiple sources such as supervisor or colleague rating to fully investigate the CCT framework.

### Future Directions

This study also highlights several future directions. First, drawing on the career construction theory (CCT) framework (Rudolph et al., 2017), we examined the mechanism and boundary conditions of proactive career behavior. According to the definition of adapting responses (Savickas and Porfeli, 2012; Hirschi and Valero, 2015; Hirschi et al., 2015; Rudolph et al., 2017), we proposed and verified that the proactive career behaviors refer to an individual taking the initiative of his or her career development tasks can be classified as adapting responses in CCT. However, scholars also proposed that adapting responses of individuals consequently influence their adaptive results (Savickas and Porfeli, 2012; Rudolph et al., 2017). Thus, we hereby call future researchers to conduct a more integrative model, which includes adaptation results, to fully understand the antecedents and consequences of proactive career behavior as well as its psychological mechanism based on the CCT (Savickas, 2002, 2005, 2013).

Second, although our finding that prevention focus did not significantly affect career adaptability is consistent with previous studies (Creed et al., 2009; Yousefi et al., 2011; Li et al., 2015; Shin and Lee, 2018), prevention focus is theoretically relevant to career adaptability and may be distant antecedent to career adaptability (Barrick and Mount, 2005; Kanfer and Chen, 2016). Therefore, future research should continue to examine and clarify with respect to the underlying mechanisms of how a prevention focus affects the career adaptability of individuals. For example, whether having a prevention focus involves adopting different mediation mechanisms for the four dimensions (i.e., concern, control, curiosity, and confidence) of career adaptability, and whether the VUCA (i.e., volatility, unpredictability, complexity, and ambiguity) context moderates the relationship between prevention focus and career adaptability.

Third, we adopted and empirically tested an integrated model by the conceptual framework of CCT, which helps to understand how and why regulatory foci of individuals enact proactive career behavior. Drawing from the CCT, we proposed and verified that job embeddedness as an individual-level contextual factor is an important moderator in influencing the generation process of proactive career behavior. However, empirical studies have revealed that organizational-level contextual factors, such as perceived organizational support (Abou Hashish, 2015), ethical work climate (Abou Hashish, 2015), job support (Lecca et al., 2020), excessive workload (Kowalczuk et al., 2020), and workplace incivility (Cash et al., 2019), would influence the work perceptions and career behaviors of the employees (i.e., job satisfaction, career satisfaction turnover intention, work-related stress, and burnout); thus, in line with the career conceptual theory, more contextual factors influencing proactive career behavior should be considered and examined within the framework of CCT to enrich and expand the antecedents and contingency roles predicting proactive career behavior. Herein, by taking the interaction impact into the grant, we call for more studies aiming to clarify and specify the mechanisms of proactive career behavior within the conceptual framework of CCT (Rudolph et al., 2017).

Last but not least, we recognize our study was conducted in one Chinese construction company, thus our results may have a limitation of samples. Future research should continue to examine and explore whether our findings can be replicated in other cultures as well as broader samples of different industries. In addition, by identifying the significance of multiple sources in collecting data, we call for more studies to examine the whole CCT framework, and highly recommend scholars to conduct such studies with multiple sources and in multiple waves in the future.

## Conclusion

We drew on career construction theory (CCT) to contribute to the understanding of why some employees exhibit career adaptability and adopt proactive career behaviors, while others do not. We found that, compared with a prevention focus, a promotion focus is a promising proactive orientation that can optimize proactive career behavior of individuals by focusing on enhancing their career adaptability, and the effect of career adaptability on proactive career behavior, which is amplified by their on-the-job embeddedness and mitigated by their off-the-job embeddedness. We hope that our study will encourage scholars to further explore the antecedents and consequences of career adaptability more comprehensively and identify the moderating factors that can likely amplify the positive effects of career adaptability.

## Data Availability Statement

The raw data supporting the conclusions of this article will be made available by the authors, without undue reservation.

## Author Contributions

All the authors were involved in the writing of the theoretical background and discussion sections. PP and YS collected the data, made statistical analyses, and wrote the manuscript. All authors contributed to the article and approved the submitted version.

## Conflict of Interest

The authors declare that the research was conducted in the absence of any commercial or financial relationships that could be construed as a potential conflict of interest.

## Publisher's Note

All claims expressed in this article are solely those of the authors and do not necessarily represent those of their affiliated organizations, or those of the publisher, the editors and the reviewers. Any product that may be evaluated in this article, or claim that may be made by its manufacturer, is not guaranteed or endorsed by the publisher.

## Footnotes

^1^To further validate our use of the shortened scale of prevention focus, we obtained a separate sample of 278 employees working at various organizations and found that the four-item shortened scale of prevention focus was highly correlated with the five-item full scale (*r* = 0.98, *p* < 0.001), and this shortened measure yielded an excellent fit for a one-factor model: χ^2^_(2)_ = 2.21 (*p* = 0.33), CFI = 0.999, TFI = 0.998, RMSEA = 0.02, SRMR = 0.01.

^2^To further validate our use of the shortened scale of off-the-job embeddedness, as we validated the shortened scale of prevention focus, we obtained a separate sample of 278 employees working at various organizations and found that the nine-item shortened scale of off-the-job embeddedness was highly correlated with the 12-item full scale (*r* = 0.99, *p* < 0.001), and this shortened measure yielded an excellent fit for a one-factor model: χ^2^_(17)_ = 22.91 (*p* = 0.15), CFI = 0.996, TFI = 0.992, RMSEA = 0.04, SRMR = 0.03.
